# Advances in Diagnosis and Treatment of Gynecological Malignancies: A Special Issue in Line with 2030 Agenda

**DOI:** 10.3390/jcm11133797

**Published:** 2022-06-30

**Authors:** Luca Roncati

**Affiliations:** Department of Surgery, Medicine, Dentistry and Morphological Sciences with Interest in Transplantation, Oncology and Regenerative Medicine, Institute of Pathology, University of Modena and Reggio Emilia, 41125 Modena, Italy; luca.roncati@unimore.it or roncati.luca@aou.mo.it or emailmedical@gmail.com

Among the 17 Sustainable Development Goals (SDG) of the United Nations 2030 Agenda, “good health and well-being” is point number 3 (SDG3), and all our efforts must be calibrated in this direction worldwide [1]; precise diagnoses and effective treatments are indispensable to achieving this important goal.

According to the Global Cancer Observatory, sponsored by the International Agency for Research on Cancer, in 2020, around the world, there were: 604.127 new cases of cervix uteri cancer and 341.831 related deaths, 417.367 new cases of corpus uteri cancer and 97.370 related deaths, 313.959 new cases of ovarian tumor and 207.252 related deaths, 45.240 new cases of vulvar malignancy and 17.427 related deaths, 17.908 new cases of vaginal neoplasm and 7.995 related deaths [2]. Therefore, cervical cancer is, today, the most frequent gynecological malignancy, burdened with the highest overall mortality. This is certainly due to the oncogenic role of the Human Papilloma Virus (HPV), toward which a broader vaccination campaign needs to be extended—in particular, toward underdeveloped and developing countries.

England has been one of the first nations to introduce routine anti-HPV vaccination (1 September 2008) for girls aged 12–13 years with a catch-up program for females aged 14–18 years in 2008–2010. As a result, anti-HPV immunization has successfully almost eradicated cervical cancer in women born after 1 September 1995 [3].

If HPV also plays a substantial role in many vaginal and vulvar malignancies, the same cannot be stated for endometrial and ovarian cancer, where genetics, lifestyle and the environment are key aspects. In fact, the reduction in illness and death from hazardous chemicals and pollution is a declared outcome target of SDG3 [4]. In conjunction with that, the impact of endocrine disruptors on hormone-sensitive female tumors should not be overlooked [5].

Ovarian cancer remains a «big killer» with the highest lethality rate among the gynecological malignancies. Massive parallel sequencing applied to solid or liquid biopsy is currently trying to reveal more and more in depth its molecular alterations for targeted therapies. In this regard, poly ADP-ribose polymerase (PARP) inhibitors (niraparib, olaparib, rucaparib) have already been approved by the Food and Drug Administration (FDA) and by the European Medicines Agency for previously treated ovarian cancer with mutations in the breast cancer (BRCA) genes [6,7,8].

In addition to the aforementioned classical tumors, there are also rarer ones, such as lymphoma and melanoma of the gynecological tract. These usually assume aggressive forms; for example, mucosal melanoma begins in a growth phase that is not horizontal and thin, as usually occurs in the skin, but vertical and thick with metastatic potential from the onset [9,10,11,12,13]. To defeat or chronicize it, great hope is placed in modern immunotherapies, such as checkpoint inhibitors (atezolizumab, ipilimumab, nivolumab, pembrolizumab) [14]. Breakthroughs in basic science enabling checkpoint inhibitor treatments have led James Allison and Tasuku Honjo to win the Nobel Prize in Physiology or Medicine in 2018 [15]. Just one year earlier, the FDA had approved the extension of pembrolizumab to any unresectable or metastatic solid tumor, gynecological malignancies included, with mismatch repair deficiency or microsatellite instability, thus making a new step forward in tissue-agnostic anti-cancer therapy [16].

Much progress still needs to be made between now and 2030 for the well-being of the female cancer population in the interest of the whole community and future generations.
